# Eugenol improves high-fat diet/streptomycin-induced type 2 diabetes mellitus (T2DM) mice muscle dysfunction by alleviating inflammation and increasing muscle glucose uptake

**DOI:** 10.3389/fnut.2022.1039753

**Published:** 2022-11-08

**Authors:** Yuge Jiang, Chuanxing Feng, Yonghui Shi, Xingran Kou, Guowei Le

**Affiliations:** ^1^Center for Food Nutrition and Functional Food Engineering, School of Food Science and Technology, Jiangnan University, Wuxi, China; ^2^The State Key Laboratory of Food Science and Technology, Jiangnan University, Wuxi, China; ^3^School of Perfume and Aroma Technology, Shanghai Institute of Technology, Shanghai, China

**Keywords:** eugenol, diabetes, glucose, spices, muscle

## Abstract

Eugenol has been used in dietary interventions for metabolic diseases such as diabetes and obesity. However, the protective effect of eugenol on muscle function in diabetes is unclear. In this study, a high-fat diet (HFD) with a streptozocin (STZ) injection induced type II diabetes mellitus in a mouse model. Oral eugenol lowered blood glucose and insulin resistance of HFD/STZ-treated mice. Eugenol reduced HFD/STZ-induced muscle inflammation and prevented muscle weakness and atrophy. Eugenol administration significantly increased GLUT4 translocation and AMPK phosphorylation in skeletal muscle, thereby enhancing glucose uptake. By silencing the transient receptor potential vanilloid channel 1 (TRPV1) gene in C2C12 myotube cells, eugenol was found to increase intracellular Ca^2+^ levels through TRPV1, which then activated calmodulin-dependent protein kinase-2 (CaMKK2) and affected AMPK protein phosphorylation. In conclusion, eugenol is a potential nutraceutical for preventing high-glucose-induced muscle impairments, which could be explained by its mediating effects on glucose absorption and inflammatory responses in the muscle.

## Graphical Abstract

Signal pathway of hypoglycemic mechanism of eugenol in the muscle of HFD/STZ-induced diabetic rats. 
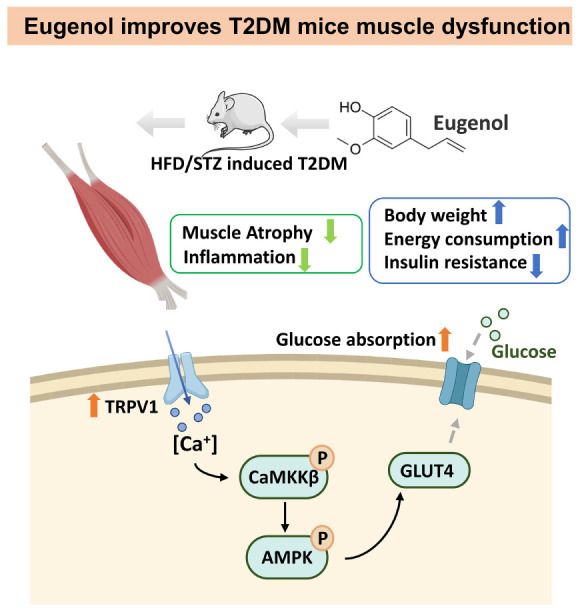

## Introduction

Eugenol, the primary component of cinnamon, has anti-bacterial and anti-inflammatory functions and helps with metabolic problems (1). Eugenol can improve lipid metabolism disorders induced by a high-fat diet (HFD) by regulating oxidative stress (2). It has been demonstrated to improve liver gluconeogenesis and regulate blood glucose homeostasis through the Adenosine 5′-monophosphate (AMP)-activated protein kinase (AMPK) signaling pathway (3). Additionally, it is often used as a surgical anesthetic and plays a role in autoregulation (4). The anesthetic effect of eugenol depends on the transient receptor potential (TRP) signaling pathway, which mainly acts on transient receptor potential V1/V4/A1 (TRPV1, TRPV4, TRPA1). These TRP family targets regulate pain perception and temperature regulation (5). However, TRPV1 regulates energy metabolism, according to recent findings (6, 7).

Transient receptor potential vanilloid 1, also known as capsaicin receptor 1, can be activated by the specific binding of compounds with a vanillin group structure, such as capsaicin (8), piperine (9), and eugenol (10). Sensory nerves that express TRPV1 innervate all organs, including the hypothalamus (11) and skeletal muscle (12). TRPV1 protects diabetic liver against hyperglycemia and hyperlipidemia *via* OPA1 (13). Piperine may also impact glucose absorption in skeletal muscle by activating TRPV1 and influencing downstream glucose transporters (14). Recent studies have shown that capsaicin activates muscle cell ATP production and glucose oxidation *via* TRPV1 rather than the insulin pathway (15).

Although eugenol can activate TRPV1, it may have the same activation or inhibitory effects as capsaicin (16). TRPV1 acts as an ion channel in the cell membrane, controlling entry and exit of Na^+^, K^+^, and Ca^2+^ (17). Ca^2+^ is a cellular regulatory messenger that is significantly associated with neural signaling (18), metabolism (19), and inflammatory responses (20). The flow of Ca^2+^ into cells may affect energy metabolism through the activation of downstream signaling pathways by calmodulin (21). On the other hand, diabetes leads to increased systemic inflammation, which in turn leads to complications (22, 23). Eugenol has been shown to regulate the level of inflammation in the body through signaling pathways such as NF-κB and COX-2 (24, 25). Therefore, in this study, we investigated whether eugenol could affect muscle function through muscle inflammation levels and glucose uptake in a high-fat-diet (HFD) plus streptozocin (STZ)-induced type 2 diabetes mellitus (T2DM) model. Furthermore, we examined the relationship between Ca^2+^ and AMPK signaling pathways and TRPV1 action *in vitro* to understand the possible mechanism of glucose regulation by eugenol.

## Materials and methods

### Animals and experimental design

A 6-week old male C57BL/6N mice were purchased from GemPharmatech (Nanjing, China). All experimental animals were kept in the Laboratory Animal Center of Jiangnan University under standardized conditions and were inspected and approved for experiments by the Animal Ethics Committee of Jiangnan University (JNU201804205).

**FIGURE 1:**
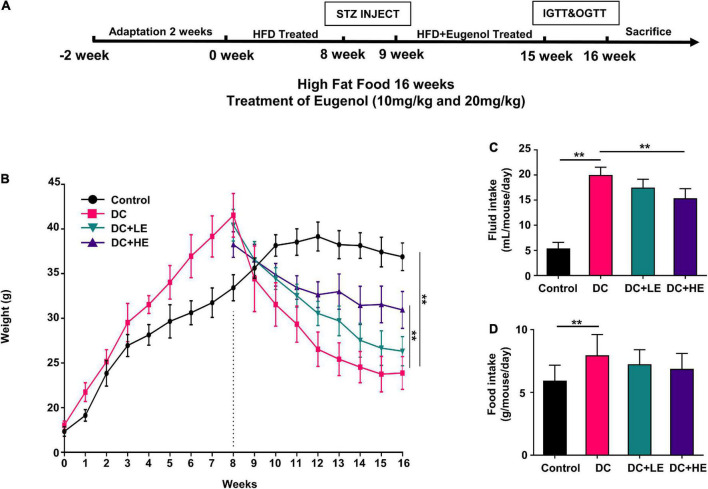
Effects of Eugenol on body weight gain of high-fat diet (HFD)-streptozocin (STZ) induced type 2 diabetes mellitus (T2DM) mice. The mice were randomly assigned into five groups: (1) control group; (2) diabetes control group (DC); (3) DC + LE group; (4) DC + HE group; **(A)** experimental schedule of Eugenol treatment; **(B)** body weight. **(C)** fluid intake. **(D)** food intake. Data are expressed as the mean ± SD, *n* = 10. *p < 0.05, **p < 0.01.

Figure 1A displays the design of the animal experiment. After 2 weeks of acclimatization, the mice were divided into normal and high-fat feeding groups. The normal group mice (*n* = 10) were fed a standard diet (LAD3001M, TROPHIC, Nantong, China), and the high fat group mice (*n* = 50) were fed a high-fat diet (HFD, 45% kcal from fat, TP23000, TROPHIC, Nantong, China) for 8 weeks. The main formula of the feed is presented in Supplementary Table 2. After 8 weeks of feeding, mice in the HFD group were examined and selected for insulin resistance. Insulin-resistant mice received STZ (Solarbio, Beijing, China), whereas the normal group received a buffer solution. STZ was diluted in a 50 mM citric acid buffer, which was administered twice at a dosage rate of 35 mg/kg body weight, with a three-day gap between each administration (26, 27). After STZ injection, mice with fasting blood glucose (FBG) levels higher than 11 mM were selected for grouping and experimentation as T2DM model. They were randomly divided into four groups (*n* = 10): (a) control group (Con, standard diet), (b) diabetes control group (DC, HFD/STZ), (c) low-dose eugenol group (DC + LE, HFD/STZ + 10 mg/kg/d eugenol), and (d) high-dose eugenol group (DC + HE, HFD/STZ + 20 mg/kg/d eugenol). Eugenol were obtained from Macklin Technology (Shanghai, China). The eugenol dose was based on previous studies and our preliminary experiment on food intake (5–6 g/d/mice) after injecting STZ. Eugenol was administered to the mice through their feed for eight consecutive weeks.

### Oral glucose tolerance test and insulin tolerance test

All animals were fasted for 8 h, and blood glucose was measured as a baseline value. Blood glucose levels were monitored at 30, 60, 90, and 120 min following glucose gavage (1.8 g/kg body weight) or insulin injection (0.8 U/kg bodyweight). The area under the curve (AUC) for glucose content calculation was used as the result for OGTT and ITT. Blood samples were collected through the tail tip of mice in a quiet state, which was then cleaned promptly to stop bleeding.

### Tissue sample collection

After completion of the OGTT and ITT experiments, the mice met the expectations of variation within the ethical specifications, and all experimental animals were executed by cervical dislocation. All blood was collected into centrifuge tubes containing heparin and centrifuged at 4°C and 3,000 × *g* for 15 min to isolate the plasma. Muscle fractions were quickly dissected and divided into gastrocnemius muscles, placed in liquid nitrogen, and finally stored in a −80°C refrigerator.

### Forelimb grip strength test

A Grip Strength Meter (Yiyan, Beijing, China) was used to test the forelimb grip strength of the mice. The mice naturally held to the rod on the dynamometer, which was moved horizontally and slowly until the mouse released the rod due to exhaustion.

### Body energy metabolism test

The body energy metabolism of mice was monitored by the crams system 1 week before slaughter. The main monitoring indicators included the 24 h respiratory exchange ratio (RER) value, autonomous activity, and energy consumption of mice. The RER value is the ratio of carbon dioxide production to oxygen consumption. Energy consumption was calculated by the following formula: Energy consumption = (3.815 + 1.232 × RER) × V_*O*2_. The amount of autonomous activity was calculated according to the number of times the infrared ray was broken in the horizontal and vertical directions. Before the formal experiment, the mice were placed for 48 h in a metabolic cage, and the data obtained through monitoring for 24 h were used for statistical analysis. During the monitoring period, the mice were free to eat and drink water, and their food intake was monitored.

### Measurement of inflammatory cytokinesody

The Elisa kit’s manufacturer’s instructions (Huijia, Xiamen, China) were followed while measuring inflammatory cytokinesody.

### Histopathology and hematoxylin and eosin staining

Fixed in 4% paraformaldehyde for 24 h, tissues were embedded in paraffin and sliced in 5 μm thick slices. TRPV1 antibody was used to stain slices for 24 h, which were treated with 3% H_2_O_2_ in methanol for 15 min. Subsequently, the slices were washed, incubated with a secondary antibody for 1 h, and examined under a microscope to measure the intensity of immunostaining. Image analysis, including muscle fiber length measurements, were performed using the Image-Pro Plus (IPP) 6.0 software.

### Cell culture and viability assay

C2C12 cells (National Collection of Authenticated Cell Cultures, Shanghai, China) were grown in an incubator with 5% CO_2_ at 37°C in Dulbecco’s modified Eagle’s medium (DMEM, Gibco, CA, USA) with 10% fetal bovine serum (FBS, Gibco, CA, USA). The manufacturer’s recommended methods for cell development were followed, and differentiation was achieved by switching the growth media to 2% horse serum, which was supplemented for 4 days.

Cytotoxicity was measured at different doses using a CCK-8 kit (Beyotime, Shanghai, China). The cells were seeded (4 × 10^3^ cells per well) in 96-well plates. After 12 h of growth, the medium was replaced and treated with various doses of eugenol (25, 50, 100, 200, and 400 μM) dissolved in DMSO (0.5%). The absorbance value of cells at 480 nm was read to calculate the cell cytotoxicity results.

### Glucose uptake and glycogen synthesis

A 2-NBDG Glucose Test Kit (ab235976, Abcam) and a glycogen assay kit (ab65620, Abcam) were used to test glucose absorption in C2C12 cells. The cells were seeded (4 × 10^3^ cells per well) in 96-well plates with 10% FBS in a 96-well black plate. After overnight incubation, cells were incubated with glucosamine (10 mM, Beyotime, Shanghai, China) for 6 h to construct the insulin resistance model. Eugenol (25, 50, 100, and 200 μM) or 0.1% DMSO was used as the control in 100 μl medium containing 100 μg/ml 2-NBDG. After 6 h of incubation, the glucose uptake and glycogen of C2C12 cells was detected using a fluorescent microplate reader (Epoch, Bio-Tek, Winooski, VT, USA) (Glucose: excitation/emission = 485/535 nm, Glycogen: excitation/emission = 535/587 nm).

### RNAi transfection

For RNAi experiments, C2C12 cells were transfected with 100 nmol/L of si-TRPV1 (sense: 5′-GCGUCUAGCUGG UUGCACACU-3′, antisense: 5′-GAGUAGCAACCGCCUU CAAGC-3′, obtained from Genepharma, Shanghai, China) using Lipo8000 (Beyotime, Shanghai, China) transfection reagent 48 h before differentiation induction.

### Intracellular Ca^2+^ detection

Each group’s intracellular calcium ion density was calculated using a Fluo-4 AM probe. The cell density was 4 × 10^6^/ml in each group. After glucosamine and eugenol treatment, 10 μmol/L of Fluo-4 AM was applied to the cells. The cells were then grown in the dark for 30 min before being rinsed regularly with PBS buffer. Cells carrying calcium ions were found and calculated for each group using flow cytometry.

### Quantitative real-time polymerase chain reaction analysis

Briefly, we extracted and measured the gene expression levels of the samples using RNA extraction kits, cDNA reverse transcription kits, as well as RT-PCR kits. All kits were obtained from Vazyme (Nanjing, China). RT-PCR was performed using Quantagene q225 (Mona, Suzhou, China). Gene primers were designed and synthesized by Genewiz (Shanghai, China) (Supplementary Table 1).

### Western-blot analysis

For western-blot experiments, all cell and tissue samples were lysed by RIPA solution (Beyotime, Shanghai, China), and protein denaturation was performed by loading buffer (Beyotime, Shanghai, China) after the protein concentration had been determined using a BCA kit (Thermo Fisher, Waltham, MA, USA). Protein samples were electrophoresed using polyacrylamide gels (Bio-Rad, Hercules, CA, USA) and transferred to a PVDF membrane by a semi-dry transfer method. After blocking, samples were treated with the target antibody for 12 h at 4°C. Antibodies were washed off after 1 h of incubation with secondary antibody. A staining kit (ECL chemiluminescence test kit, Vazyme, Nanjing, China), an Odyssey FC 2,800 imaging system (Licor, NE, USA) and the Image J software were used to analyze the results. The primary antibodies β-actin (#4970), NF-κB p65 (#8242), p-NF-κB p65 (#3033), p-AMPKα (#50081), AMPKα (#5832), and rabbit IgG were purchased from Cell Signaling Technology (Danvers, MA, USA). p-CAMKKβ (bs-6253R), CAMKKβ (bs-6253R), TRPV1 (bs-23926R), and GLUT4 (bs-0384R) were purchased from Bioss Antibodies Company (Beijing, China).

### Statistical analysis

Data are shown as the mean ± standard error of the mean (SD). GraphPad Prism 6.0 was used to test for noteworthy mean value fluctuations through one-way ANOVA. Multiple comparisons were made using Tukey’s test. A probability (*p*) value less than 0.05 indicates statistically significant differences.

## Results

### Effect of eugenol treatment on body weight in high-fat diet/streptozocin-induced mice

After 8 weeks of treatment, as depicted in Figure 1B, the DC group gained considerably more weight than the control group (*p* < 0.05). The DC group weighed considerably less than the control group following STZ administration (*p* < 0.05). During the 8-week treatment period following injection, the LE and HE groups exhibited a significant increase compared to the DC group (*p* < 0.05). At 16 weeks, no statistically differences were seen between the LE and HE groups. Significant increases in food consumption of 10.93% and water consumption of 39.81% were seen in the DC group (Figures 1C,D).

### Effect of eugenol treatment on respiratory exchange ratio, energy consumption, and activity of high-fat diet/streptozocin-induced mice

**FIGURE 2:**
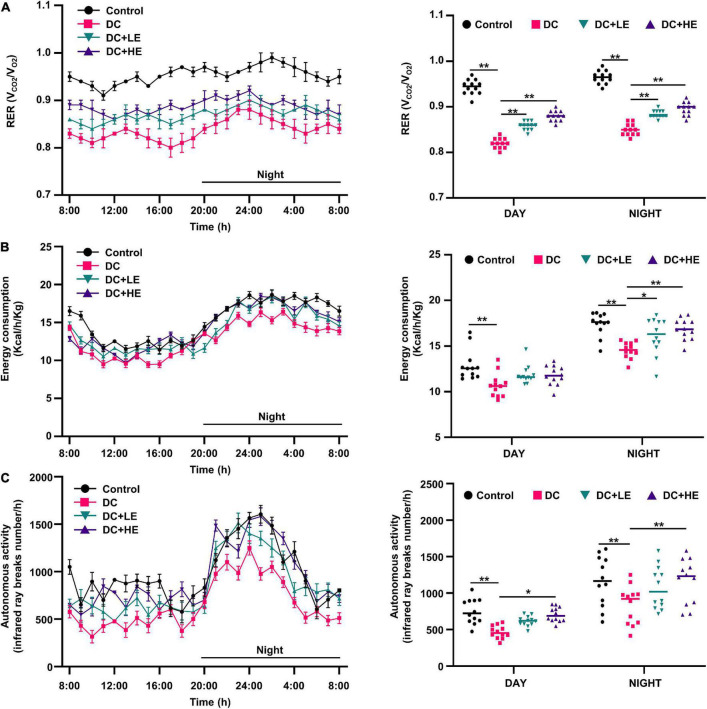
Effects of eugenol treatment on body energy metabolic status in type 2 diabetes mellitus (T2DM) mice. **(A)** 24 h respiratory exchange ratio (RER) dynamic monitoring curves of mice and mean values of daytime and nighttime respiratory exchange ratio (RER) of mice; **(B)** 24 h energy consumption dynamic monitoring curves of mice and mean values of daytime and nighttime energy consumption of mice; **(C)** 24 h autonomous activity dynamic monitoring curves of mice and mean values of daytime and nighttime autonomous activity of mice. Values for each group are expressed as mean ± SD (*n* = 10). *p < 0.05, **p < 0.01.

During 24 h monitoring, the DC group’s RER, energy consumption, and activity were lower than those of the control group (Figure 2, *p* < 0.01). During day time, the RER value of the LE and HE groups significantly increased to more than those of the DC group (*p* < 0.05), but there were no differences in energy consumption. However, at night, the activity and energy expenditure of mice after different doses of eugenol intervention were significantly higher than those of the DC group (*p* < 0.05). HE mice used 1.2 times more energy than DC mice at night.

### Effect of eugenol supplementation on insulin resistance in high-fat diet/streptozocin-induced mice

**FIGURE 3:**
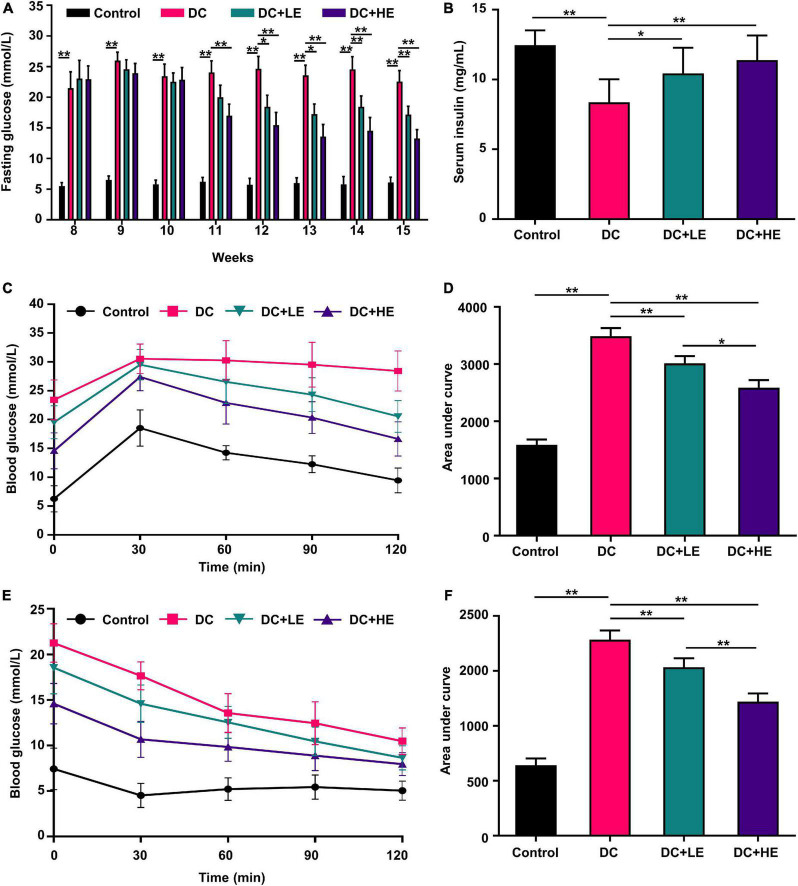
Effects of eugenol on insulin resistance of the high-fat diet (HFD)-streptozocin (STZ)-induced type 2 diabetes mellitus (T2DM) mice. **(A)** Fasting glucose level. **(B)** Serum insulin. **(C)** Oral glucose tolerance test (OGTT). **(D)** Area under the curve (AUC) analyses for Oral glucose tolerance test (OGTT); **(E)** IGTT; **(F)** Area under the curve (AUC) analyses for IGTT; **(E)** fasting glucose level. Data are expressed as the mean ± SD *n* = 10. *p < 0.05, **p < 0.01.

Oral glucose tolerance test and insulin tolerance test determined the impact of eugenol on HFD/STZ-induced insulin resistance. In the LE and HE groups, fasting blood glucose levels were 24.86 and 40.77% lower than those in the DC group, respectively (Figure 3A). The serum insulin levels were 23.52% and 36.29% higher than those in the DC group, respectively (Figure 3B). Supplementation with eugenol effectively corrected the glucose intolerance (by 26.32%) and insulin sensitivity (by 22.41%) declines in T2DM mice (Figures 3C,D). In comparison to the DC group, fasting insulin levels in the LE and HE groups rose by 28.42 and 32.0%, respectively (Figures 3E,F).

### Effect of eugenol supplementation on gastrocnemius muscle mass to alleviate weight loss in high-fat diet/streptozocin-induced mice

**FIGURE 4:**
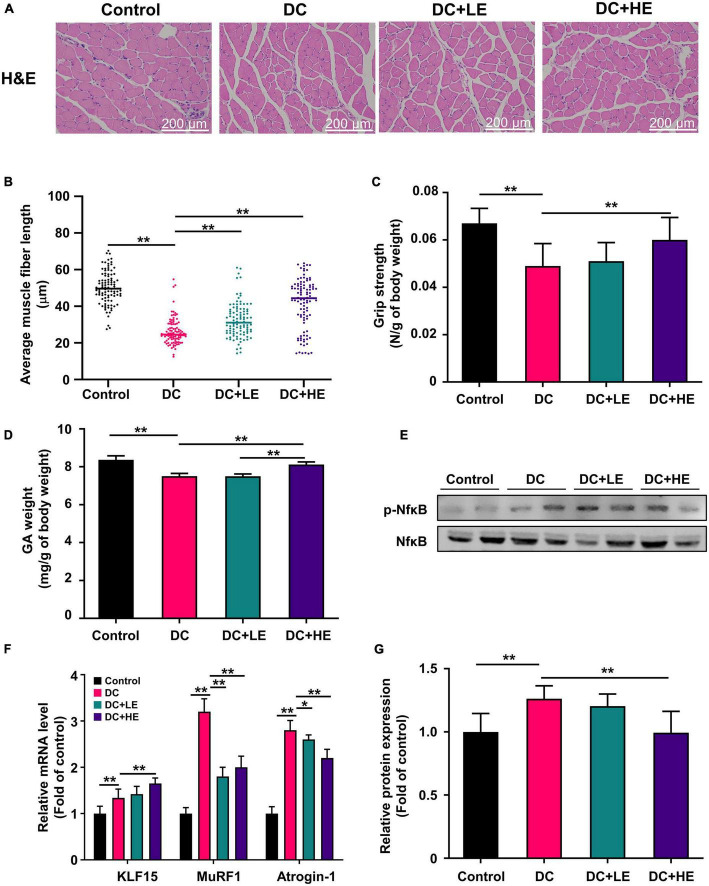
Effect of eugenol on muscle protein synthesis of the high-fat diet (HFD)-streptozocin (STZ)-induced type 2 diabetes mellitus (T2DM) mice. **(A)** H&E staining of gastrocnemius (GA) muscles section. **(B)** Grip strength was measured after 16 weeks of eugenol administration. **(C)** Effect of eugenol on gastrocnemius muscle fiber length in mice (100 samples per group). **(D)** The weights of gastrocnemius (GA) were measured right after sacrificing the mice and normalized to the body weight of each mouse. **(E)** mRNA levels of KLF15, MuRF1 and Atrogin-1 in the mice muscle. **(F)** Phospho-NFκB, NFκB, protein expression in muscle was analyzed by Western blotting. **(G)** Quantitative analysis of the Phospho-NFkB, NFkB, protein expression in muscle. Data are expressed as the mean ± SD, *n* = 10. *p < 0.05, **p < 0.01.

We assessed the grip strength and gastrocnemius (GA) mass in HFD/STZ-induced diabetic mice to assess whether eugenol had favorable effects on muscle atrophy. Figure 4B shows that the grip strength of the DC group was 20% lower than that of the control group (*p* < 0.01). Additionally, DC mice had less GA weight than the controls. GA weight in the group of HE group was significantly greater than that in the DC group (Figure 4D). Muscle function is inversely correlated with muscle fiber size (24). H&E staining was performed on muscle slices, and muscle fiber length was measured (Figure 4C). The results show that the average length of muscle fibers in the DC group was 2-fold shorter compared to the control group. The average length of muscle fibers in the LE and HE groups increased significantly by 25 and 57%, respectively, compared to the DC group (*p* < 0.01). DC + HE mice had a significantly higher GA mass and grip strength than DC mice (Figure 4C, *p* < 0.05).

### Effect of eugenol supplementation on muscle protein synthesis gene expression and inflammatory cytokines *via* NF-κB phosphorylation in high-fat diet/streptozocin-induced mice

The increase in muscle weight and reversal of muscle fiber atrophy in mice suggest that eugenol may affect protein synthesis and metabolism. We examined protein production and catabolism genes to explore protein changes. The expression levels of MuRF1 and atrogin-1 in the GA muscle increased in the DC group, and eugenol suppressed gene expression in the two dose groups (Figure 4F). Several studies have shown that muscle reduction in T2DM is associated with inflammation (28). Therefore, we measured the levels of inflammatory cytokines in the muscle (Table 1). In DC muscles, IL-1, IL-6, and TNF- levels were considerably increased than in the control group. In the HE group, the three inflammatory cytokines decreased by 45, 26, and 17%. The level of anti- inflammatory cytokine IL-10 was significantly lower in the DC group, but increased significantly in the eugenol treatment group. We also examined the protein expression of NF-κB, a transcription factor associated with inflammation. The DC group exhibited significantly higher NF-kB phosphorylation than the control group, while the eugenol group had lower phosphorylation (Figures 4E,G).

**TABLE 1:** Effects of eugenol on the levels of inflammatory cytokine in the muscle of high-fat diet (HFD)-streptozocin (STZ) mice.

	IL-1β (ng/g protein)	IL-6 (ng/g protein)	TNF-α (ng/g protein)	IL-10 (ng/g protein)
Control	5.43 ± 0.67	4.86 ± 038	46.58 ± 8.66	107.6 ± 5.83
DC	10.05 ± 0.75**	9.75 ± 0.84**	121.5 ± 7.56**	50.43 ± 4.76**
DC + LE	7.04 ± 0.54**^##^**	8.95 ± 0.73	101.4 ± 8.68**^#^**	75.35 ± 3.98**^##^**
DC + HE	6.03 ± 0.46**^##^**	7.68 ± 0.69**^##^**	96.26 ± 6.52**^##^**	89.84 ± 4.13**^##^**

**p < 0.01, compared with the control group; ^#^*p* < 0.05, ^##^*p* < 0.01, compared with the DC group.

### Effect of eugenol supplementation on skeletal muscle glycogen synthesis in high-fat diet/streptozocin-induced mice

**FIGURE 5:**
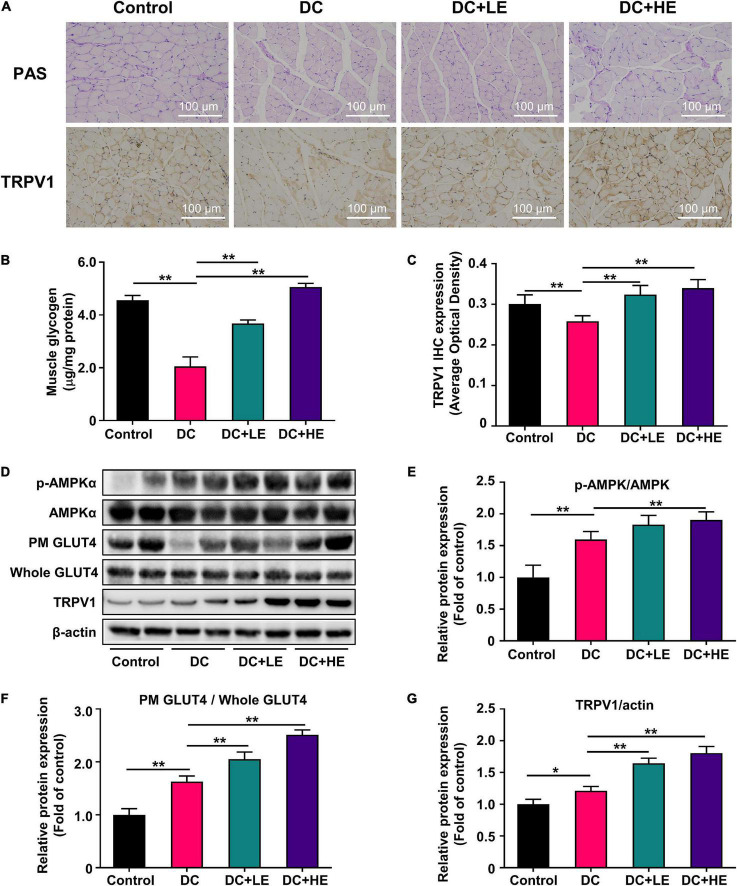
Effect of eugenol on muscle glucose metabolic of the high-fat diet (HFD)-streptozocin (STZ)-induced type 2 diabetes mellitus (T2DM) mice. **(A)** PAS staining of muscles section, and representative immunohistochemistry images for TRPV1 at 100× **(B)** muscle glycogen level of the mice. **(C)** IHC optical density score of TRPV1. **(D)** Phospho-AMPK, AMPK, plasma membrane (PM) GLUT4, Whole GLUT4 and TRPV1 protein expression in muscle was analyzed by Western blotting, with β-actin expression as an internal control. **(E)** Quantitative analysis of the Phospho-AMPK protein expression in muscle. **(F)** Quantitative analysis of the Plasma Membrane (PM) GLUT4 protein expression in muscle. **(G)** Quantitative analysis of the TRPV1 protein expression in muscle. Data are expressed as the mean ± SD, *n* = 10. *p < 0.05, **p < 0.01.

Eugenol enhances skeletal muscle protein synthesis, and to investigate whether it is associated with improved muscle glucose utilization capacity, we measured glycogen in the skeletal muscle. PAS staining of skeletal muscle and glycogen tests showed that eugenol restored T2DM-related glycogen loss and raised muscle glycogen by 20% in the HE group compared to the DC group (Figures 5A,B).

### Eugenol increases skeletal muscle glucose uptake by promoting GLUT4 displacement through the AMPK signaling pathway

We measured the expression of the glucose transporter protein GLUT4 in the plasma membrane (PM) and cell lysate (Whole). The DC group had a lower PM GLUT4 protein expression than the control group. However, the expression of PM GLUT4/Whole GLUT4 in the eugenol intervention group was 1.2-fold and 1.5-fold higher than that in DC group at low and high doses, respectively (Figures 5D,F). We also measured AMPK protein expression associated with GLUT4 displacement. T2DM reduced AMPK protein phosphorylation, but eugenol increased it by 33.3% relative to the DC group (Figures 5D,E).

### Eugenol enhances the transient receptor potential vanilloid channel 1 expression in skeletal muscle

We detected the expression of TRPV1 in the skeletal muscle by immunohistochemistry and western blotting (Figures 5C,D). Compared to the T2DM model group, eugenol increased TRPV1 protein expression in the skeletal muscle 2.4-fold. Western-blot results showed that the expression levels of TRPV1 increased by 24 and 42% after the two doses of eugenol intervention compared to the DC group, respectively (Figures 5D,G).

### Eugenol enhances glucose uptake in mouse muscle C2C12 cells

**FIGURE 6:**
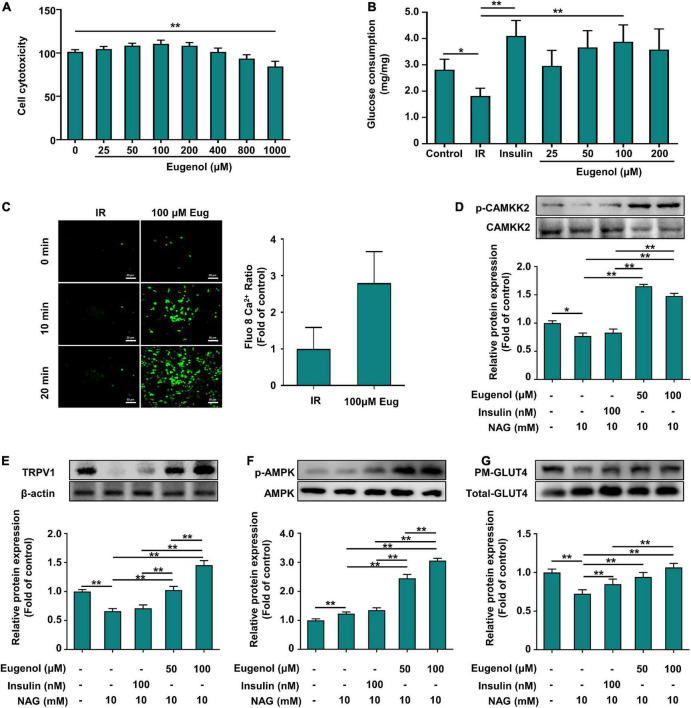
Effect of eugenol on glucose metabolic of the C2C12 cell. **(A)** Cell viability level of HEPG2 cells after 2 h of eugenol action. **(B)** Cellular glucose uptake level of C2C12 cells after 1 h of eugenol action. **(C)** Detection of Ca^2+^ levels in cells fluorescently labeled by FLUO-8AM by a spacious high-inclusion system and quantification of the results. **(D)** Phospho-CAMKK2, CAMKK2 protein expression. **(E)** TRPV1 protein expression. **(F)** Phospho-AMPK, AMPK protein expression. **(G)** Plasma membrane (PM) GLUT4 and Whole GLUT4 protein expression in C2C12 cell was analyzed by Western blotting, with β-actin expression as an internal control. Data are expressed as the mean ± SD *n* = 6. *p < 0.05, **p < 0.01.

We evaluated the effect of eugenol on glucosamine-induced insulin resistance in C2C12 cells. Eugenol showed no effect on C2C12 cell growth at 25–800 μM, whereas 1,000 μM decreased muscle cell proliferation (Figure 6A). Eugenol increased glucose uptake in cells at 25–200 μM compared with insulin resistance (IR) cells. Stimulation with 100 μM eugenol increased cellular glucose uptake approximately 1.6-fold, which was consistent with the effect of insulin stimulation in the positive control group (Figure 6B). Based on the above results, we chose a dose of 100 μM eugenol for subsequent experiments.

### Eugenol increases the inward flow of Ca^2+^ in C2C12 cells and upregulates the expression of the Ca^2+^ transport protein calmodulin-dependent protein kinase-2

TRPV1, as an ion channel protein, is associated with intra- and extracellular Ca^2+^ flow. Therefore, we measured calcium ion changes in C2C12 cells after eugenol treatment using the fluorescent probe FLUO-8 AM to label Ca^2+^ in C2C12 cells. After 20 min of eugenol treatment, intracellular Ca^2+^ influx was higher than in the control group (Figure 6C). Quantitative analysis of fluorescent images showed that intracellular Ca^2+^ content increased approximately 3-fold after eugenol intervention compared to the control group (Figure 6C).

### Eugenol increases calcium transporter protein expression *via* Ca^2+^ influx and stimulates the AMPK signaling pathway to increase glucose absorption in muscle cells

We determined the changes in the expression of CAMKK2, a calcium transport protein associated with cellular Ca^2+^ flow. The results showed that insulin stimulation did not alter CAMKK2 expression, while 50 μM eugenol intervention significantly increased CAMMK2 expression by 50% compared to the IR group (Figure 6D). We examined the phosphorylation levels of AMPK, a downstream signal of CAMKK2 that may be activated and can contribute to the displacement of GLUT4. In terms of AMPK phosphorylation expression, 100 μM eugenol, but not insulin, significantly increased AMPK phosphorylation levels 2.5-fold compared to the IR group (Figure 6F). In contrast, both insulin and eugenol increased the expression level of GLUT4 on PM, which was consistent with the glucose uptake results (Figure 6G), suggesting that eugenol and insulin differ in their mechanism of action of promoting cellular glucose uptake. Similarly, we also examined changes in TRPV1 expression and found that insulin did not alter changes in intracellular TRPV1 expression, whereas eugenol significantly increased the level of TRPV1 protein expression at different doses (Figure 6E).

### Eugenol affects glucose uptake and calcium transport in muscle cells *via* transient receptor potential vanilloid channel

**FIGURE 7:**
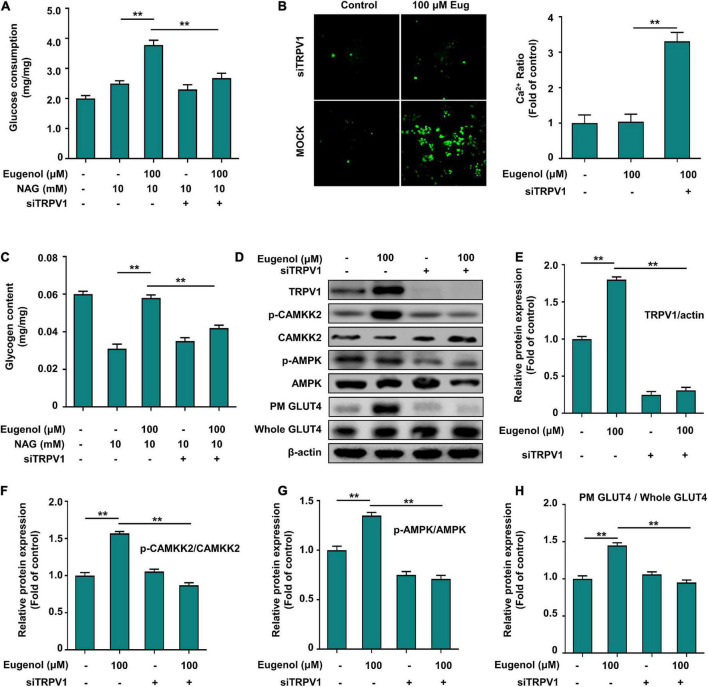
Eugenol affects glucose uptake in C2C12 cells *via* TRPV1. **(A)** Cellular glucose uptake level of C2C12 cells after 1 h of eugenol action with siTRPV1. **(B)** Detection of Ca^2+^ levels in cells fluorescently labeled by FLUO-8AM by a spacious high-inclusion system. **(C)** Cellular glycogen level of C2C12 cells after 1 h of eugenol action with siTRPV1. **(D)** The result of western-blot. **(E)** TRPV1 protein expression. **(F)** Phospho-CAMKK2, CAMKK2 protein expression. **(G)** Phospho-AMPK, AMPK protein expression. **(H)** Plasma membrane (PM) GLUT4 and Whole GLUT4 protein expression in C2C12 cell was analyzed by Western blotting after siTRPV1 and eugenol action, with β-actin expression as an internal control. Data are expressed as the mean ± SD *n* = 6. *p < 0.05, **p < 0.01.

We knocked down TRPV1 (siTRPV1) in C2C12 cells to determine if the mechanism of action of eugenol in controlling glucose absorption is connected to TRPV1 expression. The results in Figure 7A show that the effect of insulin in enhancing glucose uptake in muscle cells did not differ significantly before and after the action of siTRPV1. In contrast, muscle glucose uptake was reduced by 60% after TRPV1 knockdown with eugenol. 100 μM eugenol raised the glycogen level of IR-C2C12 cells, and siTRPV1 lowered it by 30% (Figure 7C). We used a spacious high-intensity system to capture the changes in Ca^2+^ expression and found almost no increase in intracellular Ca^2+^ in the TRPV1 knockdown group after 20 min of eugenol stimulation (Figure 7B).

### Eugenol affects Ca^2+^ inward flow through transient receptor potential vanilloid channel 1 to regulate the CAMMK2-AMPK-GLUT4 signaling pathway, thus improving glucose utilization in muscle cells

To demonstrate that eugenol affects Ca^2+^ and TRPV1-mediated downstream signaling, we determined the changes in protein expression in the signaling pathway after TRPV1 knockdown. Figures 7D,E indicate that siTRPV1 lowered TRPV1 expression, even with eugenol. The expression of the calcium transporter protein CAMMK2 decreased 3-fold after siTRPV1, even under eugenol stimulation (Figures 7D,F). The level of AMPK phosphorylation was significantly reduced after siTRPV1 and stimulation with 100 μM eugenol (Figures 7D,G), and the expression of GLUT4 on cell membranes was also significantly reduced (Figure 7D,H). These results show that eugenol may promote the displacement of GLUT4 to the cell membrane through TRPV1 by promoting inward calcium flow and activating the CAMMK-AMPK signaling pathway, ultimately leading to increased glucose uptake in skeletal muscle cells.

## Discussion

Type 2 diabetes mellitus is caused by a variety of factors, one of which is a high-fat diet that leads to decreased insulin sensitivity and, consequently, insulin resistance (29). We investigated mice treated with a high-fat diet and injected with STZ at week eight to create a T2DM mouse model. We found that the pathological symptoms of T2DM were relieved after the administration of eugenol, which led to a rapid loss of body weight in a short period owing to the inability of the mice to metabolize glucose by normal absorption and insufficient energy intake. It is likely that the diet and water intake of T2DM mice was affected by insufficient energy intake, and that the mice spontaneously increased fasting at the beginning of T2DM and increased water intake to eliminate excess glucose due to the high blood glucose level in the body (30). In this study, eugenol mitigated the weight loss associated with T2DM, and we demonstrated for the first time that eugenol enhanced water consumption, which was related with a reduction in blood glucose. Food intake did not change significantly after eugenol intervention, suggesting that eugenol does not improve T2DM symptoms in mice by restricting diet or energy intake, but may do so by enhancing energy utilization. The data of energy metabolism in mice over 24 h also demonstrated that eugenol increased the amount of energy metabolism in mice, particularly at night.

The main organs of glucose metabolism are the liver and muscles, and the liver maintains glucose homeostasis through glycolytic and gluconeogenic pathways. It has been observed that 40 mg/kg eugenol administration regulates hepatic gluconeogenesis *via* the AMPK signaling pathway, but 20 mg/kg eugenol had a limited effect on hepatic gluconeogenesis regulation (31). In the present study, we found that 10 mg/kg eugenol could still regulate fasting glucose, glucose tolerance, and insulin tolerance levels in T2DM mice. During 24 h of monitoring, eugenol increased respiratory exchange rates and energy metabolism in T2DM mice and significantly increased exercise levels at night. Skeletal muscles are an important organ for insulin action, and insulin resistance results in reduced glucose uptake, decreased exercise capacity, and eventually skeletal muscle atrophy (32, 33). It has been reported that 35% of glucose is absorbed by muscles and produces energy (34). In the absence of insulin upstream signal Akt, skeletal muscles can maintain the normal level of glucose uptake through non-insulin signaling pathways to achieve a new balance of energy uptake and metabolism (34, 35).

The fact that eugenol enhanced muscle mass in mice with T2DM prompted us to investigate its influence on muscle synthesis and catabolic signaling. Type 2 diabetes has been shown to cause muscle atrophy due to a reduction in GA fiber volume, and grip strength in mice is strongly and positively linked with muscle fiber volume (36, 37). Hyperglycemia can influence the expression of genes involved in protein breakdown and synthesis when insulin deficiency causes a rise in blood glucose levels (38). The increased expression of MuRF1 and atrogin-1 (protein degradation markers) in T2DM mice was downregulated at the mRNA level following eugenol treatment. Arogin-1 and MuRF1 gene expression levels are regulated by several transcription factors such as KLF15 and NFκB (39, 40). The intervention with eugenol had no effect on the gene expression of KLF15; however, it significantly decreased the phosphorylation level of NFκB. Studies have shown that NFκB upregulates the muscle atrophy marker MURF1 by modulating inflammation levels rather than insulin levels (FOXO pathway); therefore, we hypothesized that eugenol also improves muscle atrophy by decreasing systemic inflammation, which has been demonstrated on different animals in several previous studies (41, 42).

However, the increase in skeletal muscle protein may stem from increased energy absorption. The intervention with eugenol significantly improved glucose absorption and glycogen production in the skeletal muscle of T2DM mice. Glucose absorption could provide the energy required for muscle protein synthesis, whereas the glycogen reserves could enhance exercise capacity in mice (43). In this work, eugenol increased GLUT4 translocation to the plasma membrane *via* the AMPK route, but not the insulin pathway. Some natural compounds can regulate cellular energy metabolism through activation of downstream AMPK phosphorylation by CAMKK2 (44, 45). Our results demonstrated that eugenol intervention increased cellular Ca^2+^ inflow and CAMKK2 expression.

Transient receptor potential vanilloid channel is a ligand-gated, non-selective cation channel, whose activation leads to Ca^2+^ influx (46). Both the plasma membrane and the endoplasmic reticulum express TRPV1. It is unclear how eugenol affects TRPV1, but molecular docking experiments indicate that its vanilloid group can attach to TRPV1 as effectively as capsaicin (41). In obesity studies, activation of TRPV1 is detrimental because high fat leads to enhanced TRPV1 expression (47). Like capsaicin, eugenol may also act on TRPV1 with a dose-dependent bidirectional effect of activation followed by desensitization (48, 49). However, there are also many reports showing that TRPV1 activation can improve metabolic syndrome (50–52). Our evidence supports this view, and our results demonstrated that TRPV1 activation promotes inward calcium flow, which in turn induces enhanced muscle glucose uptake. It is possible that our dose and time of administration did not reach a desensitized state. In addition, eugenol helps restore energy metabolic homeostasis in hyperglycemic mice, as prolonged increased muscle glucose uptake is not beneficial. We hypothesized that after blood glucose levels are stabilized, eugenol changes the calcium ion flow to reach a new equilibrium, which is consistent with the mechanisms in which the brain regulates neural signaling through calcium ions (53, 54). To achieve this balance, activation and inhibition of TRPV1 as an ion channel may be a key regulatory factor.

In conclusion, we suggest that eugenol increases intracellular Ca^2+^ levels *via* TRPV1, followed by the activation of CaMKKβ and enhanced AMPK phosphorylation, leading to enhanced muscle glucose uptake and triggering an improvement in systemic energy metabolism. Thus, eugenol may be a viable phytochemical for avoiding glucose metabolism deficiencies caused by metabolic disorders.

## Data availability statement

The raw data supporting the conclusions of this article will be made available by the authors, without undue reservation.

## Ethics statement

This animal study was reviewed and approved by Jiangnan University Animal Ethics Committee.

## Author contributions

YJ and GL: conceptualization, project administration, and funding acquisition. YJ, CF, YS, and GL: methodology. YJ, CF, and GL: formal analysis. YJ, CF, and YS: investigation. YJ and CF: data curation. YJ, CF, and XK: writing—original draft preparation. YJ, CF, YS, GL, and XK: writing—review and editing. YS and GL: supervision. All authors read and agreed to the published version of the manuscript.
