# Supplementary material for: Eugenol improves high-fat diet/streptomycin-induced type 2 diabetes mellitus (T2DM) mice muscle dysfunction by alleviating inflammation and increasing muscle glucose uptake
Source: Front Nutr. 2022 Nov 8;9:1039753. doi: 10.3389/fnut.2022.1039753 (PMC9681568; doi:10.3389/fnut.2022.1039753)
Supplement: Supplementary file 1 [file Data_Sheet_1.docx]

1. Supplement

**Table S1. Sequences of primers used for RT-PCR in this study**

| **Primer sequence** | | |
| --- | --- | --- |
| Gene name | Forward primer (5’-3’) | Reverse primer (5’-3’) |
| KLF15 | GGGATCGTGGAGGAGAGCCT | CCAGCTGAGAGCTGGCTACA |
| MuRF1 | GTGTGAGGTGCCTACTTGCTC | GCTCAGTCTTCTGTCCTTGGA |
| Atrogin1 | CAGCTTCGTGAGCGACCTC | GGCAGTCGAGAAGTCCAGTC |
| GAPDH | GTCAAGGCTGAGAACGGGAA | AAATGAGCCCCAGCCTTCTC |

**Table S2. The main formula of feed**

| Materials | Standard Feed (LAD3001M)  g/Kg | High Fat Feed  (TP23000)  g/Kg |
| --- | --- | --- |
| Casein | 200 | 240 |
| Corn starch | 370 | 73 |
| Maltodextrin | 132 | 120 |
| Sucrose | 100 | 203 |
| Soybean Oil | 35 | 30 |
| Lard | 35 | 196 |
| Cellulose | 50 | 60 |
| Mineral Mix, M1021 | 59 | 59 |
| Vitamin Mix, V1010 | 12 | 12 |
| L-Cystine | 4 | 4 |
| Choline Bitartrate | 3 | 3 |
| Total | 1000 | 1000 |

Energy supply ratio of Standard Feed: Protein, 14.7%. Carbohydrate, 75.9%. Fat, 9.4%.

Energy supply ratio of High Fat Feed: Protein, 19.4%. Carbohydrate, 35.6%. Fat, 45%.

Puchased from TROPHIC Animal Feed High-Tech Co. LTD, China
